# Mediators of Neuroinflammation

**DOI:** 10.1155/2013/314261

**Published:** 2013-10-03

**Authors:** Geeta Ramesh, Mario T. Philipp, Luc Vallières, Andrew G. MacLean, Muzamil Ahmad

**Affiliations:** ^1^Division of Bacteriology and Parasitology, Tulane National Primate Research Center, Tulane University, Covington, LA 70433, USA; ^2^Axis of Neurosciences, University Hospital Center of Quebec and Laval University, Quebec City, QC, Canada G1V 4G2; ^3^Division of Comparative Pathology, Tulane National Primate Research Center, Tulane University, Covington, LA 70433, USA; ^4^Neuropharmacology Laboratory, Indian Institute of Integrative Medicine (CSIR), Sanat Nagar, Srinagar 190005, India

The understanding that neuroinflammation contributes to neurodegeneration and neuropathic pain is an emerging feature in a growing number of nervous system pathologies. Despite the immunoprivileged status of the central nervous system (CNS), its resident macrophages, microglia, with the help of other immune cells recruited from the blood, can mount robust attacks against intraparenchymal targets. Microglia also contributes to the repair of the CNS after injury and eliminates toxic molecules produced during degenerative diseases, but only modestly, possibly due to physiological adaptations that limit inflammation and its potentially devastating side effects in the CNS. Further, the complex networks of nerve cells, supportive and regulatory glial cells such as astrocytes, and oligodendrocytes in the brain and spinal cord, Schwann cells in peripheral nerves and satellite glial cells in the dorsal root ganglia as well as other cells like endothelial cells, can secrete immunoregulatory factors capable of mediating neuroinflammation. 

Although some inflammatory stimuli induce beneficial effects that help to limit disease, for instance, the killing of infectious microorganisms and elimination of damaged cells, uncontrolled inflammation may result in the production of neurotoxic factors that amplify underlying disease states. In addition, in some neuroinflammatory diseases like multiple sclerosis (MS), a breakdown of tolerance to self-antigens occurs by some unknown mechanism, leading immune cells to degrade the myelin sheath that surrounds axons. In contrast, cerebral tumor cells seem to use self-tolerance to “trick” immune cells and invade the brain tissue. After years of research in neuroimmunology, the challenge still remains of gaining a better understanding of how the activity of immune cells is regulated in the CNS, in the hope of finding a safe way to neutralize or stimulate them for therapeutic purposes.

Learning more about how inflammatory responses are induced within the nervous system and the mechanisms by which these responses ultimately contribute to pathology is fundamental in addressing the question of whether inhibition of these responses will be a safe and effective means of reversing or slowing the course of disease. It will be a challenge to design therapeutic agents that safely and effectively target only the detrimental mechanisms that contribute to disease pathogenesis. An understanding of the factors that dictate the switch from a protective to a deleterious inflammatory response will make it possible to devise interventions to limit tissue damage. 

This special issue entitled “*Mediators of neuroinflammation,*” features review articles, original research articles, and clinical studies that portray and expand the current knowledge of the specific mediators of neuroinflammation including inducers, sensors, transducers, amplifiers, and effectors of neuroinflammation. The review entitled, “*Cytokines and chemokines at the crossroads of neuroinflammation, neurodegeneration, and neuropathic pain,*” describes how cytokines and chemokines mediate neuroinflammation, with a focus on bacterial meningitis, brain abscesses, Lyme neuroborreliosis, human immunodeficiency virus encephalitis, and neuropathic pain. The protective as well as harmful effects of cytokines and chemokines are described, with an emphasis on how prolonged inflammation, continual activation and recruitment of effector cells can establish a feedback loop that perpetuates inflammation, and ultimately results in neuronal injury. 

There are two review articles that focus on the role of inflammation in ischemia in this special issue. The review entitled, “*TLR2 and TLR4 in the brain injury caused by cerebral ischemia and reperfusion,*” describes the participation of Toll-like receptors, (TLR2 and TLR4) and the resultant downstream signaling pathways that contribute to brain injury caused by cerebral ischemia and reperfusion. The review entitled “*Development and treatments of inflammatory cells and cytokines in spinal cord ischemia-reperfusion injury,*” deals with development of inflammation in spinal cord ischemia-reperfusion injury and reviews the mediators and possible treatment options. 

MS, a multifactorial neurological disease characterized by the presence of inflammatory brain infiltrates and subsequent neurodegeneration, is the focus of two review articles in this special issue. The review entitled “*Role of regulatory T cells in pathogenesis and biological therapy of multiple sclerosis,*” outlines the role of regulatory T cells in the pathogenesis and treatment of MS, while the review entitled “*MicroRNAs as novel regulators of neuroinflammation*” describes how microRNAs fine-tune the immune response in MS by functioning as crucial posttranscriptional regulators. 

The three research articles featured in this special issue focus on the therapeutic potential of certain drugs in limiting neuroinflammation, hypertension, and demyelination. The dysfunction of the blood-brain barrier (BBB) is a characteristic feature in several CNS disorders including MS. The ability of a quinolizidine alkaloid derivative called Matrine (MAT) in preventing BBB disruption in experimental autoimmune encephalomyelitis, a mouse model of MS, is described in a research article entitled “*Inhibitory effect of matrine on blood-brain barrier disruption for the treatment of experimental autoimmune encephalomyelitis,*” reporting that MAT strengthens BBB integrity by protecting the basement membrane and tight junction proteins by regulating the balance between matrix metalloproteinases (MMP2, MMP9) and tissue inhibitors of metalloproteinases (TIMP1, TIMP2). The therapeutic potential of Scutellarin, a flavone that is used in the Orient as an herbal medication, is evaluated in the research article entitled “*Scutellarin attenuates hypertension-induced expression of brain toll-like receptor 4/nuclear factor kappa B,*” in which a rat model of hypertension is used to describe the role of Scutellarin in lowering blood pressure and promoting neuroprotection by suppressing the proinflammatory TLR4/NF-*κ*B signaling pathway. The aim of the research article entitled “*Sildenafil (Viagra) protective effects on neuroinflammation: the role of iNOS/NO system in an inflammatory demyelination model*” is to study the effect of inducible nitric oxide synthase (iNOS/NO) on inflammatory demyelination and to clarify the neuroprotective effect of Sildenafil (Viagra) using the rat cuprizone model of demyelination, in which oligodendrocyte death and demyelination are independent of immune and inflammatory responses. The authors report that Sildenafil has a direct beneficial effect on oligodendrocytes, protecting these cells and improving myelination. Sildenafil also showed anti-inflammatory effects mainly through iNOS inhibition.

Inflammatory mediators are produced in Alzheimer's disease (AD) and mild cognitive impairment (MCI). Osteopontin (OPN) is a proinflammatory cytokine that has been shown to play an important role in various neuroinflammatory diseases. The clinical study featured in this special issue, entitled “*Elevated osteopontin levels in mild cognitive impairment and alzheimer's disease*”, evaluates the correlation between the levels of OPN in the cerebrospinal fluid and plasma and the cognitive deficits in the patients with AD, MCI and other non-inflammatory neurological diseases. The clinical study, entitled “*Eosinophil-derived neurotoxin is elevated in patients with amyotrophic lateral sclerosis*” is aimed at discovering a new diagnostic marker for amyotrophic lateral sclerosis (ALS), a progressive neurodegenerative disease characterized by the loss of certain motor neurons and severe spongy vacuolation of the white matter. Mediators of neuroinflammation, such as members of the family of damage-associated molecular patterns, including reactive oxygen species and eosinophil-derived neurotoxin (EDN), have been shown to play a role in the pathogenesis of ALS. A comparison of the levels of EDN in the serum samples of patients with ALS, AD, and Parkinson's disease and healthy controls revealed a 2.7-fold increase of EDN in ALS patients, suggesting that EDN could serve as a new biomarker for ALS. 

Together, the reviews, research articles, and clinical studies that are featured in this special issue enhance our knowledge base of key mechanisms in neuroinflammation.

